# Ovine Fetal Thymus Response to Lipopolysaccharide-Induced Chorioamnionitis and Antenatal Corticosteroids

**DOI:** 10.1371/journal.pone.0038257

**Published:** 2012-05-31

**Authors:** Elke Kuypers, Jennifer J. P. Collins, Reint K. Jellema, Tim G. A. M. Wolfs, Matthew W. Kemp, Ilias Nitsos, J. Jane Pillow, Graeme R. Polglase, John P. Newnham, Wilfred T. V. Germeraad, Suhas G. Kallapur, Alan H. Jobe, Boris W. Kramer

**Affiliations:** 1 Department of Pediatrics, Maastricht University Medical Center, Maastricht, The Netherlands; 2 School of Women's and Infants' Health, The University of Western Australia, Perth, Australia; 3 Department of Internal Medicine, Division of Haematology, Maastricht University Medical Center, Maastricht, The Netherlands; 4 Division of Pulmonary Biology, Cincinnati Children's Hospital Medical Center, University of Cincinnati, Cincinnati, Ohio, United States of America; University of Giessen Lung Center, Germany

## Abstract

**Rationale:**

Chorioamnionitis is associated with preterm delivery and involution of the fetal thymus. Women at risk of preterm delivery receive antenatal corticosteroids which accelerate fetal lung maturation and improve neonatal outcome. However, the effects of antenatal corticosteroids on the fetal thymus in the settings of chorioamnionitis are largely unknown. We hypothesized that intra-amniotic exposure to lipopolysaccharide (LPS) causes involution of the fetal thymus resulting in persistent effects on thymic structure and cell populations. We also hypothesized that antenatal corticosteroids may modulate the effects of LPS on thymic development.

**Methods:**

Time-mated ewes with singleton fetuses received an intra-amniotic injection of LPS 7 or 14 days before preterm delivery at 120 days gestational age (term = 150 days). LPS and corticosteroid treatment groups received intra-amniotic LPS either preceding or following maternal intra-muscular betamethasone. Gestation matched controls received intra-amniotic and maternal intra-muscular saline. The fetal intra-thoracic thymus was evaluated.

**Results:**

Intra-amniotic LPS decreased the cortico-medullary (C/M) ratio of the thymus and increased *Toll-like receptor (TLR) 4* mRNA and CD3 expression indicating involution and activation of the fetal thymus. Increased *TLR4* and CD3 expression persisted for 14 days but Foxp3 expression decreased suggesting a change in regulatory T-cells. *Sonic hedgehog* and *bone morphogenetic protein 4* mRNA, which are negative regulators of T-cell development, decreased in response to intra-amniotic LPS. Betamethasone treatment before LPS exposure attenuated some of the LPS-induced thymic responses but increased cleaved caspase-3 expression and decreased the C/M ratio. Betamethasone treatment after LPS exposure did not prevent the LPS-induced thymic changes.

**Conclusion:**

Intra-amniotic exposure to LPS activated the fetal thymus which was accompanied by structural changes. Treatment with antenatal corticosteroids before LPS partially attenuated the LPS-induced effects but increased apoptosis in the fetal thymus. Corticosteroid administration after the inflammatory stimulus did not inhibit the LPS effects on the fetal thymus.

## Introduction

Preterm birth is the leading cause of morbidity and mortality in the neonatal period [1]. In the developed world, the majority of women at risk of preterm birth receive antenatal corticosteroids to induce lung maturation and decrease infant mortality [2]. This therapy is given irrespective of the presence of an intra-uterine infection of the amniotic fluid and placental membranes (chorioamnionitis). Chorioamnionitis is present in up to 60% of preterm births and is highly associated with adverse neonatal outcomes [3]. In the majority of preterm births, chorioamnionitis is clinically silent prior to early gestational preterm labor [3]. As a result, many preterm infants are exposed to both chorioamnionitis and antenatal corticosteroids.

Exposure to intra-uterine infection may increase the risk for respiratory and neurological complications in later life [4], [5]. Intra-amniotic lipopolysaccharide (LPS)-induced chorioamnionitis causes lung [6], [7], gut [8] and skin [9] inflammation in preterm lambs, which demonstrates that chorioamnionitis causes a ‘multi-organ disease of the fetus’ [10].

The concept of fetal and early life origins of disease has developed from epidemiological studies, which correlate fetal and maternal exposures during gestation to outcomes in childhood such as asthma [11]. The pathogenesis of some diseases may result from altered T-cell immunity during fetal development [12]. The net outcome of pro-inflammation effects from chorioamnionitis and anti-inflammation effects from antenatal corticosteroids remain unstudied. As such, there is minimal information about how the fetal thymus responds to these clinically relevant exposures [13].

The thymus is the primary site for T-cell development [14]. Immature T-cells migrate from the cortico-medullary junction, move through the thymic cortex to the medullary compartment. During this migration, the immature T-cells proliferate greatly, alter antigen expression and rearrange their T-cell receptor expression [14]. Previous studies demonstrated that chorioamnionitis interferes with the development of the fetal thymus [15], [16]. In an ovine model of chorioamnionitis, Kunzmann *et al.* [17] showed that intra-amniotic LPS decreased the fetal thymus/body weight ratio and decreased thymic Foxp3 expression. However, the combined effects of chorioamnionitis and antenatal corticosteroids on fetal thymic development remain to be characterized [18].

Sonic hedgehog (Shh) and Bone morphogenetic protein (BMP) pathways participate in T-cell development and are sensitive to prenatal events such as exposure to toxins [19]–[21]. Both morphogens are produced and secreted by the thymic epithelium as negative regulators of T-cell differentiation to maintain a pool of undifferentiated, precursor T-cells in the thymus [22], [23]. We hypothesized that intra-amniotic exposure to LPS causes involution of the fetal thymus and modulation of Shh and BMP4 expression with persistent effects on thymic structure and cell populations. We also hypothesized that antenatal corticosteroids may modulate the effects of LPS on thymic development. Therefore, we exposed fetal sheep sequentially to intra-amniotic LPS and/or antenatal corticosteroids at 7-day intervals [24] and evaluated multiple indicators of thymic development. An interval of 7 days between the two interventions was chosen as representative of the interval between recognition of preterm labor and delivery for many women who deliver preterm and the probability that many early gestation fetal exposures to infection are chronic [3], [25], and that repeated administration of antenatal corticosteroids are given at weekly intervals [26].

## Materials and Methods

### Animal study

**Figure 1:**
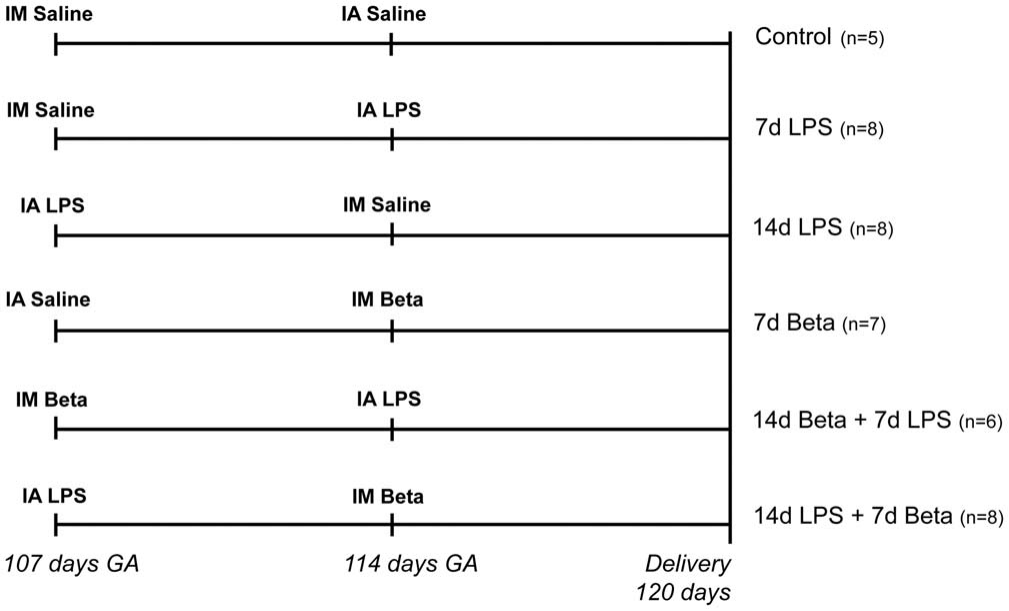
Study design. Pregnant ewes received an intra-amniotic injection of lipopolysaccharide (LPS) and/or a maternal intra-muscular injection of betamethasone (Beta) and/or an equivalent injection of saline for control animals at 107 days and/or 114 days gestation (GA). Lambs were delivered by cesarean section at 120 days GA (term = 150 days GA).

The animal experiments for this study were performed in Western Australia and were approved by the Animal Ethics Committees at The University of Western Australia (animal ethics protocol RA/3/100/830) and Cincinnati Children's Hospital Medical Center. Time-mated ewes with singleton fetuses were randomly allocated to one of six treatment groups to receive an intra-amniotic (IA) injection of lipopolysaccharide (LPS) (10 mg Escherichia Coli 055:B5, Sigma Chemical, St. Louis, MO, USA) and/or an intra-muscular injection of betamethasone (Beta) (Celestone Soluspan, Schering-Plough, North Ryde, New South Wales (NSW), Australia, 0.5 mg/kg maternal weight) and/or an equivalent injection of saline for control animals at 107 days and/or 114 days gestation (GA) (Figure 1). All ewes received a single intra-muscular injection of 150 mg medroxyprogesterone acetate (Depo-Provera, Kenral, NSW, Australia) at 100 days GA to decrease the risk of preterm birth induced by the betamethasone treatment. Despite the medroxyprogesterone acetate treatment, animals exposed to maternal betamethasone experienced fetal losses, such that we reassigned animals from a group which received betamethasone 14 days before delivery to other groups as our priority was to test the interactions of betamethasone and LPS. Lambs were delivered by cesarean section at 120 days GA (term = 150 days GA) and euthanized after birth. Intra-thoracic thymic tissue was snap frozen and fixed in 10% buffered-formalin for 24 hours. The pulmonary inflammation and maturation responses of these animals are reported elsewhere [7].

### Immunohistochemistry

Paraffin embedded thymic sections (4 µm, transverse) were stained for CD3 (DAKO A0452, DAKO Denmark), Foxp3 (eBiosciences 14-7979, eBiosciences, San Diego, USA), bone morphogenetic protein 4 (BMP4) (sc-6896, Santa Cruz Biotechnology, Santa Cruz, USA), cleaved caspase-3 (Asp175, #9661S, Cell Signaling Technology, Boston, USA) and Ki67 (Dako, M7240, DAKO Denmark). The sections were deparaffinized and rehydrated in an ethanol series. Endogenous peroxidase-activity was blocked by incubation with 0,3% H_2_O_2_ in phosphate buffered saline (PBS, pH 7.4) (for CD3, BMP4 and Foxp3) or in methanol (for cleaved caspase-3 and Ki67). Antigen retrieval was performed by incubating the sections in heated citrate buffer (10 mM, pH 6.0) for 30 minutes. Aspecific binding was blocked by incubating slides for 30 minutes with 5% bovine serum albumin (BSA) for CD3, 20% normal goat serum (NGS) for Foxp3 and BMP4 or 5% NGS for Ki67. This step was omitted for cleaved caspase-3. Slides were incubated overnight at 4°C with the diluted primary antibody (CD3 1∶200, Foxp3 1∶30, BMP4 1∶500, cleaved caspase-3 1∶400, Ki67 1∶50) followed by incubation with a secondary goat-anti-mouse (for Foxp3 and Ki67) or swine-anti-rabbit (for CD3, BMP4 and cleaved caspase-3) biotin labeled antibodies. The immunostaining was enhanced with Vectastain ABC peroxidase Elite kit (PK-6200, Vector Laboratories, Burlingame, USA) followed by a nickel sulfate-diaminobenzidine (NiDAB) staining. Sections were counterstained with 0.1% Nuclear Fast Red.

Evaluation was performed by light microscopy (Axioskop 40, Zeiss, Germany) with LeicaQWin Pro v.3.4.0 software (Leica Microsystems, Germany). CD3, Foxp3, Ki67 and BMP4 positive staining were measured in three to five representative sections at 200× magnification by Image J software (Rasband, W.S., Image J US National Institutes of Health, Bethesda, Maryland, USA). Cleaved caspase-3 positive cells were counted in three representative high power fields at 200× magnification by a blinded observer and averaged per animal.

The morphology of the thymus was evaluated by light microscopy after hematoxylin and eosin staining. The cortico-medullary (C/M) ratio was quantified for three representative sections from each animal at 2.5× magnification using Image J software (Rasband, W.S.) [27].

### RNA extraction and real-time PCR

Total RNA was extracted from frozen thymic tissue using the SV Total RNA Isolation system (Z3100, Promega, Madison, USA) according to the manufacturer's instructions. Genomic DNA contamination was removed by treatment with RQ1 DNase (M610A, Promega) and the RNA was tested for the presence of genomic GAPDH. Total RNA was reverse transcribed with the First Strand cDNA synthesis kit (4379012001, Roche-Applied, Mannheim, Germany) according to manufacturer's instructions using anchored oligo-primers. Primers for real-time PCR (RT-PCR) were constructed based on published ovine or bovine cDNA sequences (Table 1). RT-PCR reactions were performed in duplicate with the LightCycler 480 SYBR Green I Master mix (4707516001, Roche-Applied) on a LightCycler 480 Instrument according to the manufacturer's instructions. RT-PCR results were normalized to ovRSP15, a housekeeping gene, and mean fold changes in mRNA expression were calculated by the ΔΔCt-method [28].

**Table 1:** Primers used for RT-PCR.

Gene		Sequence (5′-3′)	Amplicon size	T^m^	Accession code (RefSeq)
TLR2	Fw Rv	GGCTGTAATCAGCGTGTTCA GATCTCGTTGTCGGACAGGT	160 bp	64°C	NM_001048231.1
TLR4	Fw Rv	GAGAAGACTCAGAAAAGCCTTGCT GCGGGTTGGTTTCTGCAT	200 bp	65°C	NM_001135930.1
Shh	Fw Rv	ACTGGAGCGGACCGGCTGAT CCGGCCACTGGCTCATCAC	82 bp	68°C	XM_614193.3
BMP4	Fw Rv	ACCACGAAGAACATCTGGAG TTATACGATGAAAGCCCTGC	173 bp	61°C	NM_001110277.1

### Data analysis

Groups were compared using one-way ANOVA with Dunnett's or Tukey's test for post-hoc analysis or by a non-parametric Kruskal-Wallis test as appropriate. Statistical analysis was performed by GraphPad Prism v5.0. Significance was accepted at p<0.05.

## Results

### Thymic cortico-medullary ratio

**Figure 2:**
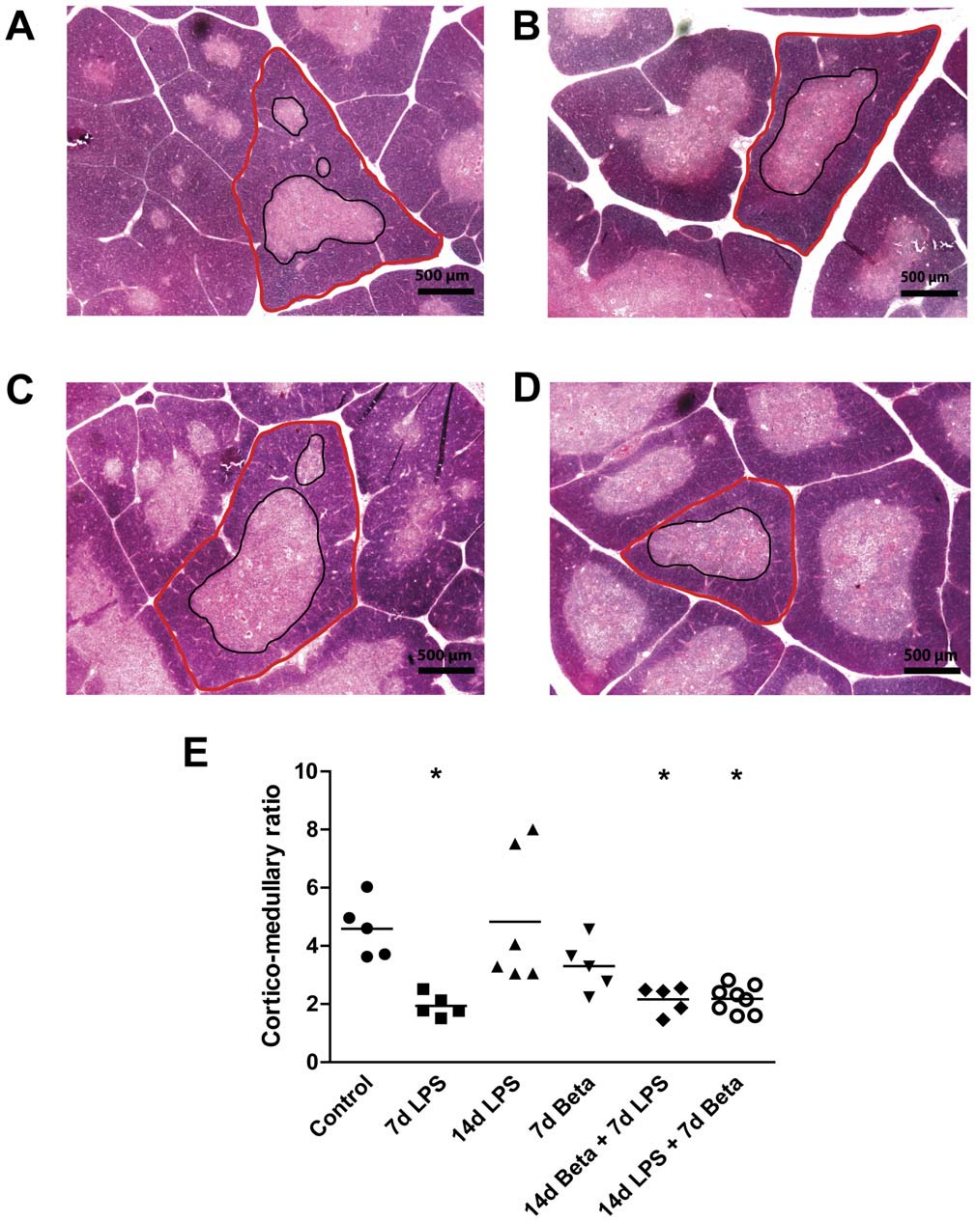
Cortico-medullary ratio. The cortico-medullary (C/M) ratio of the thymus was measured using H&E sections. Representative images are shown for controls (**A**), 7 d LPS (**B**), 14 d betamethasone (Beta)+7 d LPS (**C**) and 14 d LPS+7 d Beta group (**D**). **E:** The C/M ratio decreased in the 7 d LPS group and the combined LPS and Beta groups. Red circled area: cortex, black circled area: medulla. Magnification: 40× * p<0.05 versus controls.

The cortico-medullary (C/M) ratio decreased significantly after exposure to LPS for 7 days (Figure 2B) compared to controls (Figure 2A). Betamethasone treatment 7 days prior to LPS exposure did not attenuate the change in C/M ratio (Figure 2C). Animals exposed to LPS 14 days before delivery and then exposed to betamethasone had a reduced C/M ratio (Figure 2D) compared to controls.

### Proliferation and apoptosis

**Figure 3:**
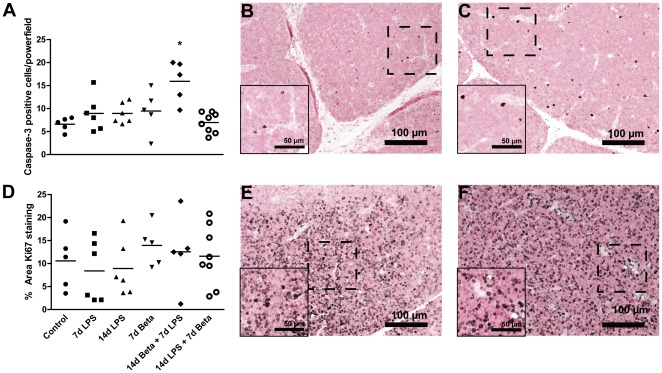
Cleaved caspase-3 and Ki67 expression. **A:** Cleaved caspase-3 positive cells increased in the thymus in the 14 d Beta+7 d LPS group (**C**) when compared to controls (**B**). **D:** No changes in the percentage of Ki67-positive stained area were detected in any of the experimental groups compared to control. Representative images are shown for controls (**E**) and 7 d LPS animals (**F**). Magnification 200×; magnification insert: 400×.* p<0.05 versus controls.

Cleaved caspase-3 positive cells, an indicator of apoptotic cells, increased in the thymus of animals which received betamethasone prior to the LPS exposure when compared to controls (Figure 3A). No changes in Ki67 expression were detected in any of the experimental groups compared to control (Figure 3D).

### TLR expression in the fetal thymus

**Figure 4:**
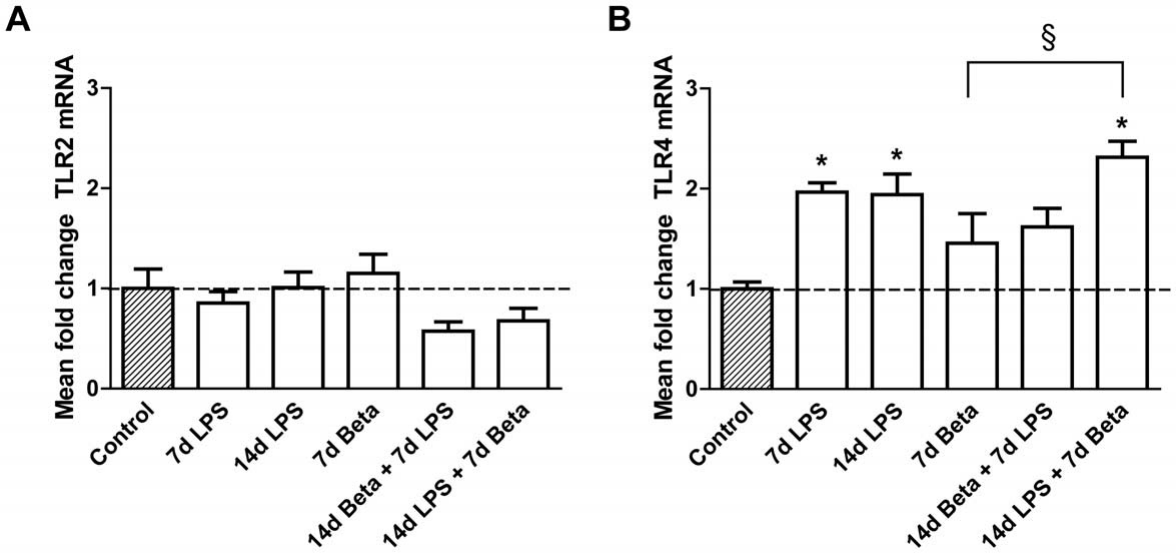
Expression of Toll-Like Receptors (TLR) 2 and 4. *TLR2* (**A**) was not differently expressed in experimental groups compared to controls. *TLR4* (**B**) mRNA increased in the 7 d LPS, 14 d LPS and the 14 d LPS+7 d Beta group. * p<0.05 versus controls and § p<0.05 between experimental groups.

*TLR2* mRNA levels did not change in the experimental groups compared to control (Figure 4A). *TLR4* mRNA almost doubled in animals which were exposed to LPS either 7 or 14 days before delivery (Figure 4B). Treatment with betamethasone 7 days after the LPS exposure did not attenuate the rise in *TLR4* mRNA levels.

### CD-3 positive thymic T-cells

**Figure 5:**
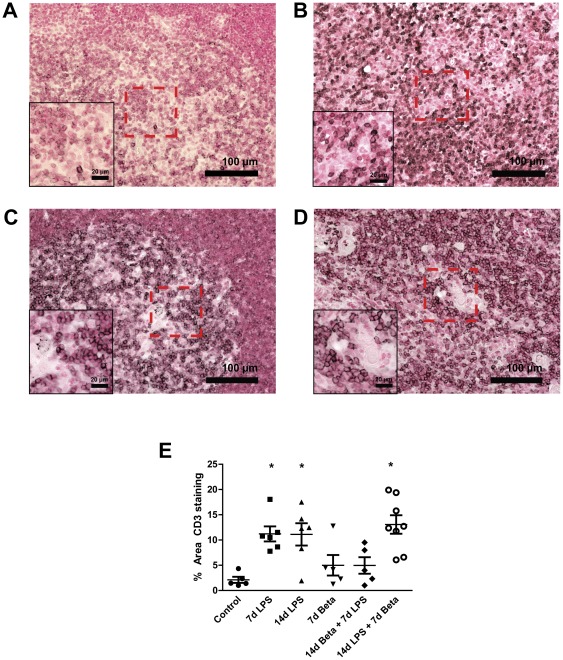
CD3-positive cells in the thymus. The percentage of CD3-positive stained area in the thymus was evaluated by immunohistochemistry. Representative images are shown for controls (**A**), 7 d LPS (**B**), 14 d LPS (**C**) and 14 d LPS+7 d Beta group (**D**). **E:** The percentage of CD3-positive area increased in the 7 d LPS, 14 d LPS and 14 d LPS+7 d Beta group. Magnification 200×; magnification insert: 400×. * p<0.05 versus controls.

The percentage of CD-3 positive stained area increased significantly 7 days (Figure 5B) and 14 days (Figure 5C) after LPS exposure compared to controls (Figure 5A) and was primarily located in the thymic medulla (Figure 5C). Betamethasone treatment after the 14 day LPS exposure did not attenuate the LPS-mediated increase in the percentage of CD3-positive stained area of the thymus (Figure 5D).

### Decreased Foxp3 expression in response to LPS

**Figure 6:**
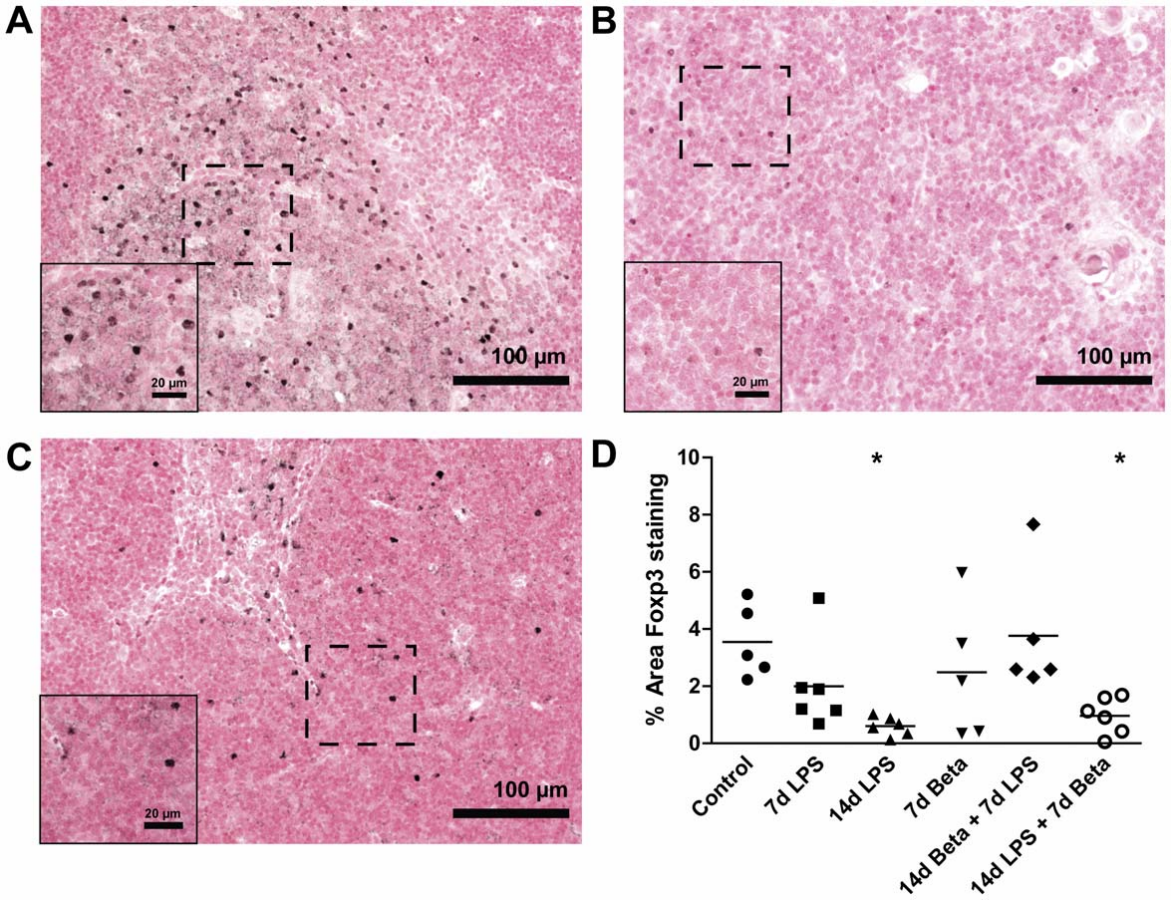
Foxp3-positive cells in the thymus. Representative images for Foxp3 expression in the thymus are shown for controls (**A**), 14 d LPS (**B**), and 14 d LPS+7 d Beta group (**C**). **D:** The percentage of Foxp3-positive stained area in the thymic medulla decreased in the animals exposed to 14 days of LPS independent of Beta treatment. Magnification 200×; magnification insert: 400×. * p<0.05 versus controls.

The percentage of Foxp3-positive stained area detected primarily in the medulla, was decreased significantly 14 days after LPS exposure (Figure 6B) compared to controls (Figure 6A) irrespectively of betamethasone post-treatment (Figure 6C). Other experimental groups did not show a change in thymic Foxp3 expression.

### Shh and BMP4 expression in the thymus

**Figure 7:**
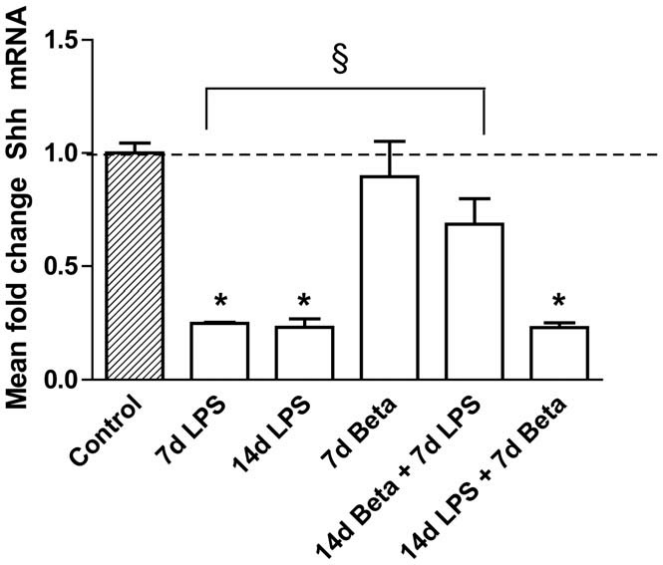
Sonic Hedgehog (Shh) mRNA expression. The mRNA levels of *Shh* were significantly decreased after 7 d and 14 d LPS exposures and in the 14 d LPS+7 d Beta group. * p<0.05 versus controls and § p<0.05 between experimental groups.

**Figure 8:**
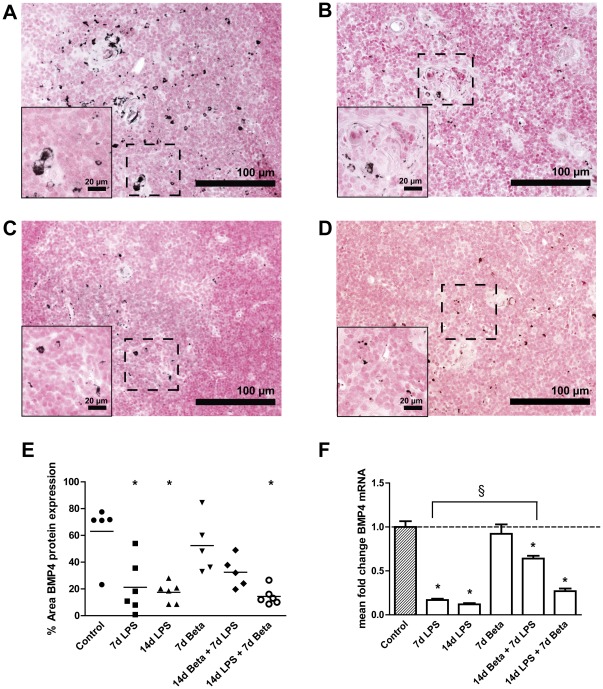
Bone morphogenetic protein 4 (BMP4) expression. Representative images for BMP4 expression in the thymus are shown for controls (**A**), 7 d LPS (**B**), 14 d LPS (**C**) and 14 d LPS+7 d Beta group (**D**). **E:** BMP4 protein expression decreased in the 7 d and 14 d LPS and the 14 d LPS+7 d Beta groups. **F:** *BMP4* mRNA levels decreased 7 and 14 days after LPS exposure irrespective of betamethasone treatment. Magnification 200×; magnification insert: 400×. * p<0.05 versus controls and § p<0.05 between experimental groups.

*Shh* mRNA (Figure 7) decreased to about 20% of the control value 7 and 14 days after LPS exposure. Similarly, BMP4 (Figure 8) mRNA and protein expression also decreased significantly 7 and 14 days after exposure to LPS. Betamethasone treatment before the exposure to LPS attenuated the decrease in *Shh* mRNA levels and BMP4 protein. *BMP4* mRNA but not protein expression remained decreased in this group compared to controls. The animals which received betamethasone treatment after the LPS exposure still had decreased levels of *Shh* and BMP4 which were similar to the LPS effect alone.

## Discussion

We investigated the responses of the fetal thymus to chorioamnionitis and antenatal corticosteroids, fetal exposures which are common prior to very preterm delivery [1], [3]. We found that intra-amniotic exposure to LPS activated the fetal thymus as shown with increased *TLR4* mRNA levels and CD3 expression, decreased Foxp3-positive cells and altered thymic structure.

In organ cultures of the fetal thymus, blocking of Shh signaling accelerated T-cell differentiation [29] while additional Shh protein arrested T-cell development [30]. Cortical epithelial cells of the thymus also produced BMP4 which controls early T-cell development [31]. Inhibition of the BMP4 signaling cascade was required for further differentiation of T-cells at several checkpoints during development [32]. *Shh* and BMP4 expression decreased in response to LPS-induced chorioamnionitis indicating increased differentiation of thymic T-cells. This increased differentiation was reflected in an increase in CD3 expression, which is expressed on mature T-cells [33]. Taken together these results indicate that exposure to intra-amniotic LPS resulted in differentiation of T-cells with an accumulation of mature T-cells in the medulla and depletion of early progenitor T-cells in the cortex, which was consistent with the changed thymic structure.

Although the involution response of the fetal thymus has been described in several human and animal studies [15], [27], the mechanistic changes behind this response remain unclear. Kunzmann et al. [17] showed an acute thymic involution with changes in Foxp3-positive cells in an ovine model of chorioamnionitis up to 5 days after exposure to LPS. Here, we further characterized this process by demonstrating that the effects of LPS on the thymic population and structure were detected 14 days after the LPS exposure and were not due to changes in proliferation or apoptosis. A persistent increase in medulla area due to the accumulation of mature, differentiated T-cells may explain the change in thymic structure.

Based on the anti-inflammatory properties of antenatal corticosteroids, a reduced inflammatory response after exposure to LPS was expected [34]. Corticosteroids can exert anti-inflammatory effects by upregulation of the IκB family, which are cytoplasmic inhibitors of NF-κB, and by direct antagonism between the glucocorticoid receptor and NF-κB, resulting in blocked transcription of responsive genes. However, in our study betamethasone administration after the inflammatory stimulus did not reverse the LPS-induced increase in *TLR4* and CD3 in the fetal thymus. LPS has a half-life of 1.7 days in the amniotic fluid and was still detectable 15 days after intra-amniotic injection [35]. Because of the slow clearance, LPS may induce a persistent inflammatory response which is in line with measurements of pulmonary inflammation in these animals [7].

Surprisingly, betamethasone administration 7 days before LPS exposure attenuated activation with no signs of inflammation in the fetal thymus. Thymic structure changed slightly due to the pro-apoptotic properties of antenatal corticosteroids [36]. Previous reports demonstrated only inhibitory effects of corticosteroids on the immune system for a maximum of 48 hours [37], [38]. Our results indicate that the antenatal corticosteroids used clinically can potentially desensitize the fetal immune system and attenuate a response to LPS. Paradoxically, these ‘longer term’ inhibitory effects of corticosteroids on the fetal immune system did not occur in the animals that were exposed to LPS and then betamethasone 7 days later as the immune system remained activated. Corticosteroids are potent immune-modulatory hormones which can have long term effects on the HPA-axis and subsequently on the function of the immune system [39], [40] which may be reflected in the unresponsiveness of the fetal immune system to LPS after corticosteroid pre-treatment. The longer-term effects of the changes in cell composition and activation of the fetal thymus after exposure to antenatal corticosteroids may depend on the timing of the exposure and the developmental stage of the immune system and therefore remain to be further determined.

Although administration of antenatal corticosteroids to pregnant women at risk of preterm birth is one of the most effective and important therapies in perinatal medicine, concerns remain about effects on fetal growth and development of the brain and immune system. Antenatal corticosteroid treatment can change the population and function of cord blood lymphocytes of preterm infants [41], [42] and may induce thymic involution [43]–[45]. Antenatal dexamethasone also was associated with decreased T-cell numbers in the fetal rat thymus and spleen and changes in the CD4/CD8 ratio [40], [46]. Dexamethasone treatment of neonatal rats changed the peripheral T-cell repertoire and altered endogenous corticosterone production of thymic epithelial cells during neonatal life [47]. These changes may impair the functional maturity of the neonatal immune system and could contribute to the increased incidence and adverse outcome of infections [48], [49].

Our findings contribute to the current concept that events during fetal life can potentially alter the function of the immune system [50]. The clinical associations between chorioamnionitis and adverse outcomes in later life such as BPD [12] or asthma [51] may be mediated in part by changes in immune responses.

In summary, our results demonstrate that fetal exposure to intra-amniotic LPS activated the fetal thymus which was accompanied by structural changes. Treatment with antenatal corticosteroids before LPS partially attenuated the LPS-induced effects but increased apoptosis in the fetal thymus. Corticosteroid administration after the inflammatory stimulus did not inhibit the LPS effects on the fetal thymus. However, insights into the effects of LPS and corticosteroids on molecular pathways such as BMP4 and Shh are limited. Due to the low expression BMP4 and a lack of specific reagents for Shh protein for ovine tissue, we were not able to perform more detailed analysis of these pathways. Further analysis at different time intervals of exposure are necessary to better understand the interactive effects of chorioamnionitis and corticosteroids on the fetal thymus. Although the design of the study does not allow us to evaluate the dynamics of the changes induced by LPS and corticosteroids, this report illustrates the complicated interactions of pro- and anti-inflammatory stimuli on the development of the fetal immune system.
